# 698. Prevalence of *Clostridioides difficile* Infection in Patients with Isolated Glutamate Dehydrogenase (GDH) Positive

**DOI:** 10.1093/ofid/ofad500.760

**Published:** 2023-11-27

**Authors:** Tanadhol Pharnusopon, Natthakit Chavapradit

**Affiliations:** Bhumibol Adulyadej Hospital, Saimai, Krung Thep, Thailand; Faculty of Medicine Siriraj Hospital, Mahidol University, Bangkok, Krung Thep, Thailand

## Abstract

**Background:**

Clostridioides difficile infection associated diarrhea is one of the world's health problems. The diagnosis method varies across guidelines. Nowadays, PCR for Clostridioides difficile is the standard modality in many institutions. But for resource-limited settings, a combination of GDH and Toxin A/B could be used. However, when an isolated positive GDH test with negative for toxin A/B, stool PCR should be done to confirm the diagnosis in highly suspected cases. This study aims to study the need for PCR testing by identifying the prevalence rate and specific characteristics associated with Clostridium difficile infection among isolated GDH-positive patients.

**Methods:**

The prospective study collected data from patients who were admitted to Bhumibol Adulyadej Hospital, a tertiary hospital center in Bangkok, Thailand, from December 2021 to April 2023. The eligible criteria were patients over 18 years of age who were clinically suspected of Clostridioides difficile infection with stool GDH positive and toxin A/B negative. After informed consent, stool PCR for Clostridioides difficile was done, and patients characteristics, treatment, and outcomes were collected. The outcomes were to study the prevalence of Clostridioides difficile infection and identify characteristics among patients that helped predict the infection.

**Results:**

There were 37 participants included in this study; 17 were male, and the median age was 63 years old. 5.4% of participants would be classified as having a fulminant Clostridioides difficile infection and 35.1% as having a severe infection if a PCR test was positive. The prevalence of PCR-confirmed Clostridioides difficile infection was 8 of 37 (21.6%). Factors that helped predict the infection included ICU admission (sensitivity 50%, specificity 79.3%), hypoalbuminemia (sensitivity 87.5%, specificity 10.3%), stool leukocytes (sensitivity 25%, specificity 65.5%), blood leukocytosis (sensitivity 37.5%, specificity 68.9%), and empirical treatment from a physician (sensitivity 50%, specificity 37.9%).

**Conclusion:**

Among patients who have stool Clostridioides difficile GDH positive but Toxin A/B negative, even though the incidence rate of disease is low, stool PCR should be done in every case due to the lack of clinically specific characteristics.

**Disclosures:**

**All Authors**: No reported disclosures

